# Comparison of Appendicitis Inflammatory Response (AIR) and Lintula scoring systems in diagnosing acute appendicitis among children

**DOI:** 10.25122/jml-2021-0049

**Published:** 2022-04

**Authors:** Mehdi Alemrajabi, Morteza Khavanin Zadeh, Sam Zeraatian-Nejad Davani, Fazil Nasiri, Sevda Riazi, Mohammad Nasiri

**Affiliations:** 1. Department of Colorectal Surgery, Firoozgar Hospital, Iran University of Medical Sciences, Tehran, Iran; 2. Department of General Surgery, Rasool-e-Akram Hospital, Iran University of Medical Sciences, Tehran, Iran; 3. Department of Obstetrics & Gynecology, Istanbul University, Istanbul, Turkey; 4. South Health Center of Tehran University of Medical Sciences, Tehran, Iran

**Keywords:** Appendicitis, Lintula, AIR, pediatric patients

## Abstract

Appendectomy is still the best treatment for acute appendicitis in pediatric patients. Given the problems of early and immediate diagnosis of acute appendicitis, defining the best diagnostic protocol for this condition is of utmost importance. Different diagnostic methods, such as Lintula and appendicitis inflammatory response (AIR) scoring systems, are used for this purpose. This study aims to compare Lintula and AIR scoring systems among children with suspicion of acute appendicitis regarding their postoperative outcomes. During two years, a prospective multicentric study was carried out in the selected hospitals of Iran. Pediatric patients admitted with the diagnosis of acute appendicitis were enrolled in the study. Before decision making, each patient's score was calculated according to two appendicitis scoring systems of Lintula and AIR. The clinical outcomes and diagnosis of patients were then compared to the results of each scoring system. For those patients who were a candidate to undergo surgery, the final diagnosis of acute appendicitis was made by histopathology. Patients were divided into high and low-risk groups according to scoring systems outcomes. Among the patients with lower scoring for appendicitis, the AIR scoring system had a sensitivity and specificity of 95%, which was more promising than that of the Lintula system (19%); however, the specificity was comparable between the two models (74% *vs.* 83%). For patients at higher risk of acute appendicitis, although the AIR scoring systems did not provide reliable results (sen: 45% and spe: 25%), the Lintula scoring showed remarkable sensitivity (87%), accompanied by a high diagnostic accuracy (87%). AIR and Lintula scoring systems are not accurate models to predict the risk of acute appendicitis among children; therefore, they can serve as an adjacent modality for other diagnostic methods.

## Introduction

Acute appendicitis has been known as the most common surgical emergency during childhood, being the leading cause of acute abdominal pain that demands surgical removal of the appendix [1]. Delay in the diagnosis and treatment of appendicitis might result in serious and life-threatening complications, such as perforation that develops in up to 75% of the patients and wound infection, which might impact up to 11% of the victims. Therefore, timely treatment through surgical resection has been considered the gold standard in treating acute appendicitis for decades [2, 3]. Although many efforts were made to acquire an early and accurate diagnosis in patients suspected of acute appendicitis, the histopathological evaluation of the post-appendectomy specimen remains the only reliable test to confirm the diagnosis [4, 5]. Accordingly, the rates of complicated acute appendicitis are still far from ideal, and even significant advancements in the diagnostic evaluation of children with suspected appendicitis have not changed the management of this condition. Thus, the demand for a feasible and meticulous diagnostic technique during the preoperative phase remains intact.

For decades, clinical manifestations were utilized to establish the diagnosis of appendicitis, including the Lintula scoring system. However, the high rate of false-positive outcomes resulted in unnecessary appendectomies in several patients. Therefore, the paraclinical workups, including laboratory tests and imaging modalities, were used to determine the inflammation and get an accurate diagnosis. Several scoring systems and models have recently turned out to play an essential role in establishing the early and accurate diagnostic in individuals' suspicion of acute appendicitis, including the AIR and Lintula scoring systems [6, 7]. These scoring systems and algorithms were introduced to decision-making through the clinical management of the patients, albeit the postoperative histopathology examinations remain the gold standard modality in the diagnosis of the patients. Thus, diagnosis according to clinical and paraclinical findings, utilizing them among pediatric patients remains challenging due to the diversity of the clinical manifestations [8, 9]. Various studies have compared the role of the scoring tools in categorizing adult patients with acute abdomen. Nonetheless, there is still a significant controversy regarding the use of these tools, particularly in pediatric patients, since very little knowledge is available about this population. 

To our knowledge, few studies have evaluated the role of diagnostic tools in the discrimination of treatment approach among children presented with acute appendicitis. This study aimed to compare the accuracy of Lintula and AIR scoring systems in the diagnosis of acute appendicitis among pediatric patients.

## Material and methods

The present prospective multicentric study was performed under the Shahid Beheshti University of Medical Sciences, Tehran, Iran, from October 2016 to October 2017. All pediatric patients admitted with the suspicion of acute appendicitis were evaluated, and patients who underwent surgical intervention were enrolled in the study. Patients suffering from generalized peritonitis and those with previous intra-abdominal surgery, were excluded. 

All demographic and clinical information, including body temperature and fever, characteristics of abdominal pain, such as pain intensity, pain relocation or migration, nausea and vomiting, anorexia, tenderness, rebound tenderness, guarding, bowel sounds, total white blood cell counts, and differentials were collected using a questionnaire. The standard gold diagnosis was considered the histopathology outcomes of the patients after surgical intervention. Prior to patients' examination, attending physicians or surgery residents trained on the two appendicitis scoring systems of Lintula and AIR, and the cut-off points for diagnosis calculated the patient's risk of appendicitis [10, 11]. The two scoring systems' diagnostic and evaluation criteria are listed in Table 1.

**Table 1.** Clinical scoring systems in the management of suspected appendicitis in children.

			**Lintula**	**AIR**
**Male gender**			2	-
**Symptoms**		Nausea/Vomiting	2	
		Nausea		1
		Anorexia		
		Migration of pain to RLQ	4	
**Signs**	Pain in RLQ		4	1
		Severe	2	
		Mild to moderate	0	
	Rebound tenderness		7	
		Mild		1
		Moderate		2
		Severe		3
	Guarding		4	
	BT>37.5°C		3	
	BT>38.5°C			1
	Absent bowel sounds		4	
**Laboratory tests**	Leukocytosis shift			
	PMN Leukocytosis	70–84%		1
		>85%		2
	WBC count	>10 x 109		
		10–14.9 × 109		1
		>15 × 109		2
	CRP concentration	10–49 g/L		1
		>50 g/L		2
**Risk of Appendicitis**		Low risk	0 – 15	0 -- 4
		Intermediate risk	16 -- 20	5 -- 8
		High risk	21 -- 32	9 -- 12

AIR – Appendicitis Inflammatory Response; RLQ – Right Lower Quadrant; BT – Body Temperature; PMN – Polymorphonuclear; WBC – White Blood Cell; CRP – C-Reactive Protein.

All patients underwent abdominal ultrasonography or abdominal computed tomography scan to evaluate the intra abdominal inflammation and appendix. Subsequently, the attending physician decided on the patients' management. The decision was made based on the clinical outcomes and diagnosis of patients and compared to the results of each scoring system. For those patients who were a candidate to undergo surgery, the final diagnosis of acute appendicitis was made by histopathology. According to the clinician's decision, patients who did not have appendicitis were discharged and prescribed analgesics. 

### Statistical analysis

To evaluate the diagnostic accuracy of the scoring systems compared to postoperative diagnosis, the sensitivity, specificity, positive predictive value (PPV), and negative predictive value (NPV) were calculated. Receiver operating characteristics (ROC) curve analysis was carried out. The analysis was restricted to two distinct subgroups for each scoring system based on the patients with a lower and higher risk of acute appendicitis, considering the different cut-off points defined for each scoring system to distinguish the risk of appendicitis. The significance level was set at 0.05, and all results were expressed by frequency (percent) for qualitative variables and Mean±SD (standard deviation) for quantitative variables. All analyses were carried out using SPSS version 25.

## Results

The current study was carried out among 661 children with abdominal pain and suspicion of acute abdomen. Of these, 265 patients (40%) were male, and 396 (60%) were female, with a mean age of 8.9±3.44 years. None of the patients with acute abdomen were missed during the initial evaluation at the emergency room. On 389 children (52%), diagnostic imaging was performed. The most frequent alternate diagnosis was nonspecific abdominal pain (184 (57.8%) patients), followed by gastroenteritis (in 97 (30.5%) patients), chronic constipation (26 (8.1%) patients), and intussusception (11 (3.4%) patients). Subsequently, 343 (51.8%) patients underwent surgical intervention. During the intraoperative phase, the surgical evaluation revealed no evidence of appendicitis in 31 patients (9%), and an unnecessary appendectomy was carried out. Accordingly, 312 patients underwent acute laparoscopic or open laparotomy. According to postoperative histopathologic assessment, acute appendicitis and phlegmonous appendicitis were detected in 238 (76.3%) and 74 (23.7%) patients, respectively. 

In Table 2, the sensitivities, specificities, PPVs, and NPVs of the Lintula and AIR scoring systems in predicting appendicitis probability were summarized with due attention to the severity of signs and symptoms. Among patients with scores of lower appendicitis risk, the analysis revealed that the AIR scoring system benefits better sensitivity in the diagnosis of acute appendicitis, with the rate of 95%, even though the Lintula scoring system has a considerably lower sensitivity rate (19%) compared to AIR scoring. However, based on the higher specificity rate (83%) of the Lintula scoring system in terms of acute appendicitis diagnosis, AIR (specificity rate: 74%) seemed to be less effective in detecting patients without acute appendicitis. In addition, the accuracy of the Lintula system was higher than that of the AIR system (83% *vs.* 74%).

**Table 2.** Diagnostic accuracy of the scoring systems based on patients' risk of developing appendicitis.

		**Sensitivity (CI95%)**	**Specificity (CI95%)**	**Positive Likelihood Ratio (PLR) (CI95%)**	**Negative Likelihood Ratio (NLR) (CI95%)**	**Positive Predictive Value (PPV) (CI95%)**	**Negative Predictive Value (NPV) (CI95%)**	**Accuracy**
**AIR**	**High**	48% (42.13–52.95)	25% (20.19–29.97)	0.63 (0.56–0.72)	2.11 (1.70–2.62)	0.16% (0.14–0.18)	99.47% (99.35–99.58)	25%
	**Low**	95% (91.83–96.86)	74% (69.04–78.93)	3.67 (3.04–4.44)	0.07 (0.04–0.11)	0.91% (0.76–1.10)	99.98% (99.97–99.99)	74%
**Lintula**	**High**	18% (14.85–20.93)	87.5% (67.64–97.34)	1.42 (0.49–4.14)	0.94 (0.80–1.10)	0.35% (0.12–1.03)	99.76% (99.73–99.80)	87%
	**Low**	19% (13.86–25.64)	83% (79.44–86.38)	1.14 (0.80–1.63)	0.97 (0.90–1.05)	0.29% (0.20–0.41)	99.76% (99.74–99.78)	83%

Although the Lintula scoring system had a similarly lower sensitivity rate (18%) in diagnosing acute appendicitis in patients with higher severity scores, the specificity of 87.5% was noticeable in distinguishing the patients with a lower probability of acute appendicitis. Despite the slightly higher sensitivity rate (48%) produced by AIR, the specificity rate (25%) was considerably lower than that of the Lintula scoring system. Despite an extremely high NPV, both scoring models had an interestingly low PPV. However, the Lintula (accuracy: 87%) scoring model could significantly outperform the AIR (accuracy: 25% (CI95%=21.65% to 28.38%)) scoring system in predicting appendicitis in pediatric patients who had higher scores, representing a higher probability of acute appendicitis.

## Discussion

Scoring systems were developed to accelerate and assure a reliable estimate of the risk of appendicitis by evaluating the different clinical and paraclinical findings, including clinical symptoms, physical exam findings, and laboratory data [3]. Consequently, the diagnostic accuracy increases by maximizing the diagnostic information and considering it individually. In this study, we aimed to investigate the accuracy of the two well-known scoring tools used for clinical management of appendicitis in patients with acute abdomens. Therefore, the Lintula and AIR scoring systems were compared in a prospective cohort considering the operative outcomes of the patients. Only a few studies have compared the diagnostic accuracy of the different scoring systems in ruling out appendicitis in children. Thus, the discriminatory power of these scoring systems and their influence on clinical outcomes has not been widely discussed. To our knowledge, this is one of the first studies to prospectively compare the accuracy of AIR and Alvarado scores in estimating appendicitis development in pediatric patients [5, 12–14]. The main strength of the study is the assessment of the scoring systems with due attention to the confirmed postoperative diagnosis, which reflects the main criterion for diagnosis of acute appendicitis in pediatric patients. In addition, this study benefits of a multicentric design by including several hospitals with many cases. 

In this study, an AIR score of less than five had a negative likelihood ratio of less than 0.1, which resembles its impact on clinical decision-making. However, in terms of patients with scores of more than six, the AIR system fails to guarantee an accurate and reliable diagnosis, with due attention to its low sensitivity and specificity and very low accuracy. Therefore, the AIR system might not be ideal for evaluating patients with a high probability of acute appendicitis, as it carries a higher risk of missing these patients. Furthermore, despite its high accuracy, the Lintula system suffers from a low sensitivity, which leads to high debate over its efficacy. 

On the other hand, a meta-analysis that evaluated the accuracy of the less than five Alvarado score in ruling out appendicitis revealed a pooled sensitivity of 99% and specificity of 43% [15]. Although the overall sensitivity and specificity were slightly greater than 80%, a remarkable inconsistency was reported among pediatric patients with appendicitis [16–18]. However, in our study, AIR and Lintula scoring systems represented a similar and even higher accuracy in patients with lower scores regarding the probability of acute appendicitis. With due attention to its higher sensitivity (95%) in detecting the patients with a lower risk of appendicitis, the AIR score may be preferable in pediatric patients. In addition, considering the factors playing a role in the decision-making process based on Lintula scoring, including physical signs and symptoms, such as nausea, migration, and severity of pain, this system could not be able to address the clinical manifestations in pediatric patients, which might have led to the extremely low sensitivity of the Lintula scoring in our study [17]. Despite adult patients, the main weakness of the Lintula system in evaluating pediatric patients might contribute to the lack of laboratory tests evaluation in this system [17, 20]. Therefore, it can be stated that, despite its low accuracy, the AIR system might be a favorable choice for Lintula in diagnosing children with appendicitis. However, we believe none of the above-mentioned risk scores benefit sufficient sensitivity and specificity in the effective diagnosis of appendicitis as a solitary modality [21]. On this basis, it can be hypothesized that scoring systems can only be used as an adjunct screening modality to initially rule out the risk of appendicitis in children and might help diminish the risk of negative appendectomy. However, the necessity of additional diagnostic modalities, such as laboratory tests and imaging, is not neglectable. The appendicitis scoring systems might be used in future studies comparing their ability to rule out appendicitis in children presenting with abdominal pain at the emergency department. 

However, our study has some limitations regarding the definition of symptoms and their onset characteristics as it might be difficult for children to evaluate pain intensity and severity of guarding. In addition, physicians' judgment in evaluating the clinical manifestations could be uncertain, particularly in pediatric wards. Finally, due to the prospective design of the investigation, we could not evaluate any cases of missed appendicitis in the current study since all patients were followed up until their complete recovery.

## Conclusion

The AIR and Lintula scoring systems cannot be used as a solitary modality for distinguishing patients with acute appendicitis, considering the less reliable diagnostic accuracy. Therefore, a specific pediatric scoring system and criteria are needed to meticulously rule out appendicitis in children with suspicion of acute appendicitis.

## Acknowledgments

### Conflict of interest

The authors declare no conflict of interest.

### Ethical approval

This study was approved by the Organizational Committee of Ethics in Biomedical Research of Shahid Beheshti University of Medical Sciences (IR.SBMU.970215.17, October 2015).

### Consent to participate

Written informed consent was taken from the parents of every patient who participated in this study after explaining the procedures.

### Authorship

MA contributed to conceptualizing the study. MKZ contributed to the methodology, and MN contributed to writing the original draft. SZD contributed to editing the manuscript. FN contributed to data collection. SR contributed to data curation and data analysis.
